# Radon Spa Therapy for the Treatment of Musculoskeletal Disorders: A Systematic Review and Meta-Analysis

**DOI:** 10.7759/cureus.101547

**Published:** 2026-01-14

**Authors:** Dimitrios P Christakos, Ioannis Alatsathianos, Mira Moebel, Monif Morshed Salah, Philip Manolopoulos, Ioannis Karnezis, Sailesh Mahapatra, Andreas Papadopoulos, Stavroula Eirini Gantzoula, Michael-Alexander Malahias

**Affiliations:** 1 Orthopaedics and Traumatology, KAT Attica General Hospital, Athens, GRC; 2 Epidemiology and Biostatistics, School of Medicine – Frankfurt, European University Cyprus, Frankfurt am Main, DEU; 3 Orthopaedics and Traumatology, School of Medicine – Frankfurt, European University Cyprus, Frankfurt am Main, DEU; 4 Orthopaedics and Traumatology, Penteli Children’s Hospital, Athens, GRC

**Keywords:** clinical outcomes, musculoskeletal disorders, non-pharmacological treatment, pain reduction, radon spa therapy

## Abstract

Emerging evidence indicates that radon spa therapy can confer beneficial effects on pain alleviation and quality of life in individuals with musculoskeletal disorders (MSDs). This meta-analysis aims to systematically synthesize the available literature examining the clinical outcome of radon spa therapy on patients with MSDs. A search of PubMed, MEDLINE, Scopus, Google Scholar, and the Cochrane Database of Systematic Reviews was performed using the Preferred Reporting Items for Systematic Review and Meta-Analyses from the inception of the database to June 2025. Studies were included if they reported outcomes for pain intensity, functional capacity, or quality of life/health status using validated measures after treatment with radon spa. Overall, eight studies met the inclusion criteria with 2,152 patients and a mean of 7.9 ± 2.2 months of follow-up. The mean Modified Coleman Score was 70.25 (out of 100), indicating a good methodological quality. The meta-analysis of pain intensity revealed significant effects of radon spa treatment versus controls in the medium to long-term period. The pooled effect size difference was 0.2720 (95% confidence interval (CI) = 0.1204 to 0.4237, p = 0.0004), with low heterogeneity (I² = 43.28%, Q = 6.87, p = 0.1431). For the overall radon spa treatment effect compared to baseline, the medium to long-term period showed a pooled Hedges’ g of 0.9936 (95% CI = 0.0801 to 1.9070, p = 0.033), indicating a statistically significant therapeutic effect of radon spa treatment in patients with MSDs. There is good-quality evidence to show that radon spa therapy is significantly better compared to placebo in reducing pain at the medium to long-term follow-up of patients suffering from MSDs. Based on these results, radon spa therapy can be considered an effective alternative to other conservative means in the management of MSDs.

## Introduction and background

Musculoskeletal disorders (MSDs) are the leading cause of disability and incapacity for work. Approximately 1.71 billion people worldwide already suffer from these conditions [1]. The prevalence of MSDs is expected to rise continuously in the following years due to the increase in life expectancy and the sedentary lifestyle of the Western population. People suffering from MSDs have reduced ability to participate in social life, they are often forced to resign early from their jobs, and they have an overall worse quality of life (QoL) compared to the rest of the population [2].

The treatment strategies of MSDs encompass conservative and operative means of treatment. Conservative management measures comprise both pharmacological and non-pharmacological interventions. Pharmacological measures include local analgesics, painkillers, nonsteroidal anti-inflammatory drugs (NSAIDs), and corticosteroids. Non-pharmacological choices include weight loss, exercise and physiotherapy, orthotics, and surgery in severe cases [3,4]. These treatment options aim to reduce pain, regain range of motion, improve functionality, and delay the disease process [5]. Many of these disorders, however, require lifelong treatment, making the development of cost-effective and safe treatments essential. For example, NSAIDs, COX-2 selective inhibitors, and opioids may be effective in reducing pain in MSDs, but they have been associated with serious side effects such as gastrointestinal bleeding, cardiovascular adverse events, opioid-induced hyperalgesia, opioid tolerance, and opioid addiction [6].

Spa therapy is one of the oldest non-medical therapies used for the treatment of MSDs, which is of value in both degenerative and inflammatory arthritis [7,8]. Radon spa therapy for MSDs has been an alternative for the management of the clinical symptoms of MSDs, including pain, physical disability, and poor QoL [9]. Although there are several studies examining the clinical outcome of radon spa in the treatment of MSDs, there is no systematic analysis in the contemporary literature to examine its efficacy in improving the clinical outcome of MSDs [10-17]. For this reason, we conducted a meta-analysis to assess the potential therapeutic role of radon spa in the treatment of MSDs. Our primary hypothesis was that radon spa treatment improves clinical outcomes of patients with MSDs by significantly reducing pain in the short-, mid-, and long-term period.

## Review

Methodology

Search Criteria

This research was conducted in accordance with the Preferred Reporting Items for Systematic Reviews and Meta-Analyses (PRISMA) guidelines [18]. The US National Library of Medicine (PubMed/MEDLINE), Scopus, Google Scholar, and the Cochrane Database of Systematic Reviews were queried for publications utilizing the following keywords: “radon” AND “spa” AND “therapy” OR “treatment,” “radon” AND “balneotherapy,” “radon” AND “hot spring” OR “hot springs,” “radon” AND “bath” OR “baths” OR “bathing,” “radon” AND “thermal” AND “baths” OR “spring” OR “baths” OR “bathing” OR “springs” since inception of database to June 2025. No limit was set regarding the year of publication. Two authors (MM and SM) independently conducted all the searches and screened the titles and abstracts to identify relevant studies. Only abstracts that reported clinical outcomes for patients treated with radon spa using validated measures were included. If the title and abstract of each study contained insufficient information, the full manuscript was reviewed. An additional search was conducted by screening the reference list of each selected article. When studies were reporting outcomes on the same cohort of patients, the one with the longest follow-up was selected.

Study Selection and Data Extraction

Studies were included if they reported outcomes for pain intensity, functional capacity, or QoL/health status using validated measures after treatment with radon spa. Only papers published in the English language were included in the study. Studies that were still ongoing were excluded. The quality assessment of the studies for methodological deficiencies, as a common alternative to risk of bias, was examined by the modified Coleman Methodology Score [19]. To ensure methodological transparency and compliance with reporting standards, we performed a risk of bias evaluation using the Cochrane Risk of Bias 2.0 (RoB 2) tool [20] for randomized control trials and the Risk of Bias in Non-randomized Studies of Interventions (ROBINS-I) tool [21] for non-randomized studies. The level of evidence in the included studies was determined using the Oxford Centre for Evidence-Based-Medicine Level of Evidence (LoE) [22]. Pain intensity was assessed using the Numeric Rating Scale (NRS), Visual Analog Scale (VAS), and other standardized pain scores. Functional capacity was measured with tools such as the Keitel Functional Index [23]. QoL/health status was evaluated using the EQ-5D index [24], EQ-VAS [24], and Arthritis Impact Measurement Scales [25].

Data Preparation and Composite Scores

The treatment groups were defined as radon spa therapy and control interventions involving any water-based sessions (including artificial CO_2_ baths, natural mineral water, or other non-radon treatments), with the control group explicitly excluding radon in the water. Results were stratified into four categories based on the time points at which the effects of radon therapy were measured in the studies included in this review. Thus, the early-term period refers to results measured till four weeks after treatment, the short-term period till 12 weeks, the mid-term period till 24 weeks, and the long-term period till 36 weeks or more. For studies that did not explicitly report mean values and standard deviations, the WebPlotDigitizer tool was utilized to extract these metrics from graphical plots. The standardized mean difference (SMD), expressed as Hedges’ g, was calculated for pre- and post-intervention scores, assuming a correlation coefficient of 0.5 between baseline and post-intervention variances to account for within-study variability. For studies reporting multiple outcomes (e.g., different pain scales, functional capacity measures, or QoL indices) at the same time point, composite Hedges’ g scores were computed using the formula:

Composite Hedges’ $g = \frac{\sum g_i}{n}$

where gi​ represents the individual effect sizes, and n is the number of studies. The composite variance (Vg) was calculated as:

$V_g = \frac{1}{n^2} \left( \sum V_{g_i} + 2 \sum r \sqrt{V_{g_i} V_{g_j}} \right)$

where r = 0.5 is the assumed correlation coefficient between measures, and the second term adjusts for covariances among paired effect sizes. This approach standardized effect sizes across heterogeneous outcome measures within each study.

Meta-Analysis

A meta-analysis was performed using a random-effects model with the restricted maximum likelihood estimator to pool Hedges’ g effect sizes across studies. Variance estimates were derived from study-specific standard deviations, sample sizes, and the computed Vg values. Percentage change in outcomes was calculated as:

$\text{Percentage Change} = g \times \frac{\text{Baseline SD}}{\text{Baseline Mean}} \times 100$

where g is the Hedges’ g effect size.

Heterogeneity was assessed using the Ι^2^ statistic, with values >50% indicating substantial heterogeneity, and the Q statistic with its associated p-value to test for significance.

Sensitivity Analyses

A leave-one-out analysis was conducted to evaluate the robustness of the results. This involved iteratively removing one study at a time and recalculating the pooled effect size to assess whether any single study disproportionately influenced the overall results.

Forest Plots and Percentage Change

Forest plots were generated to visualize Hedges’ g effect sizes and their 95% confidence intervals (CIs) for each time point (immediate-to-short term, medium-to-long term) and outcome that showed statistical significance. Plots for the overall radon effect (versus baseline) and radon versus control comparisons were ordered by study year, with the null effect (g = 0 or g_diff = 0) marked. For radon versus control plots, “Control” and “Radon” labels were added below the x-axis to clarify treatment arms.

Statistical Analysis

All analyses were conducted in R (version 4.4.1) using the metafor and dplyr packages. The significance level was set at p-values <0.05.

Results

**Figure 1:**
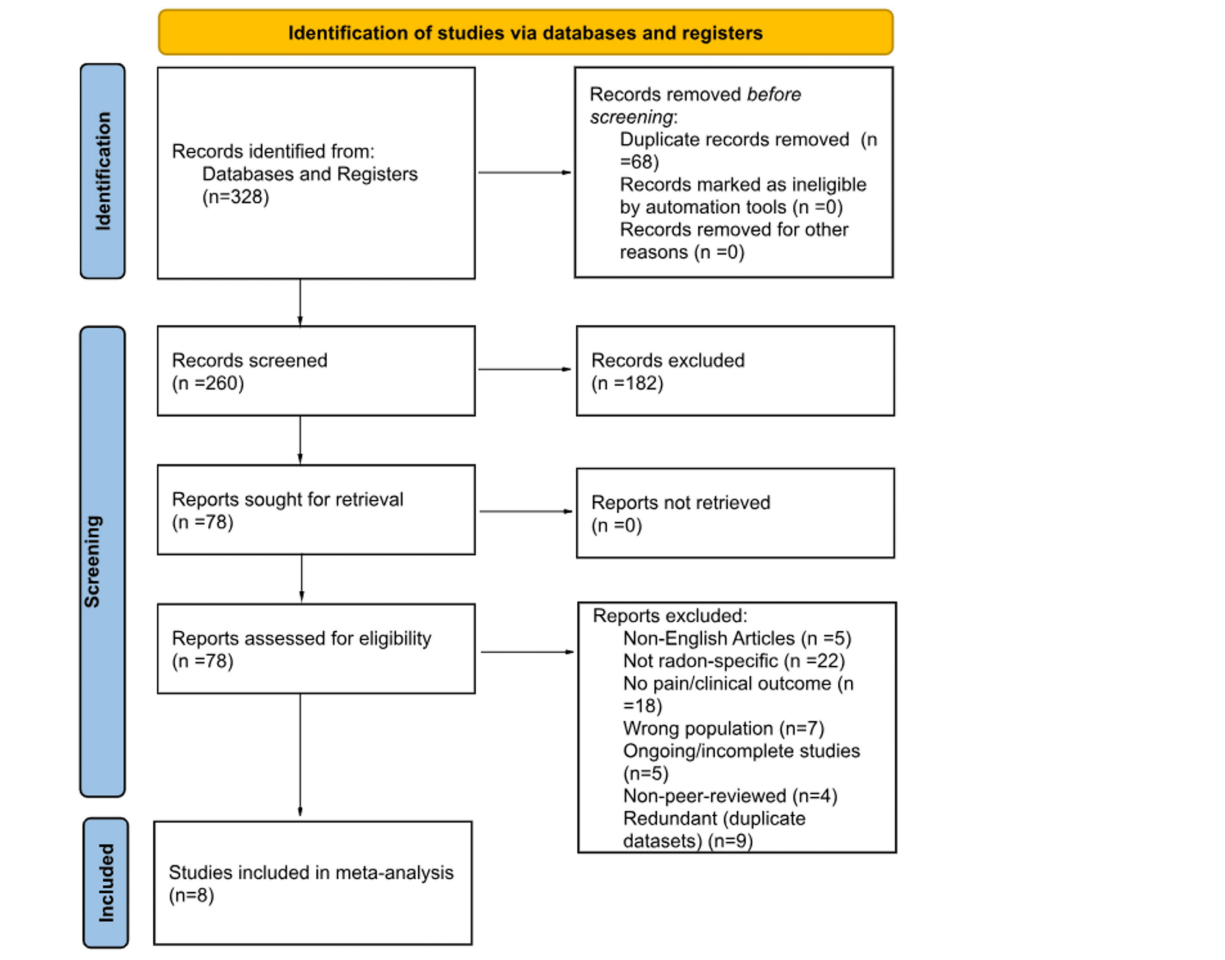
Preferred Reporting Items for Systematic Reviews and Meta-Analyses (PRISMA) flow diagram demonstrating the systematic review process.

The literature search initially yielded 328 relevant citations (Figure 1). After removal of duplicate studies, 260 were subject to application of the predetermined inclusion and exclusion criteria. Following the application of these criteria, 78 studies were subject to a full-text screening process. Ultimately, eight studies were included for analysis [10-17] (Table 1).

**Table 1:** Studies included in the meta-analysis.

Author (year)	Type of study	Modified Coleman Score	Patients initially enrolled	Patients who completed the study	Sex, male/female	Age (years), mean (range)	Follow-up (months)	Level of evidence
Donaubauer et al. (2024) [10]	Prospective randomized placebo-controlled trial	80	116	91	43/73	59 (39–75)	6	II
Van der Zee-Neuen et al. (2023) [14]	Retrospective observational studies of prospectively collected data	66	561	512	286/275	55	9	ΙΙΙ
Van der Zee-Neuen et al. (2022) [15]	Retrospective observational studies of prospectively collected data	63	469	291	163/128	51.8 (19–79)	9	ΙΙΙ
Gaisberger et al. (2021) [16]	Prospective, randomized control pilot study	61	28	25	10/15	67.36 (60–75)	6	II
Rühle et al. (2018) [17]	Prospective, observational study	60	103	97	37/60	59.9 (28–75)	6	IIΙ
Annegret et al. (2013) [11]	Prospective, randomized, blinded, multi-center clinical trial	80	681	652	263/389	58.3	9	ΙΙ
Franke et al. (2007) [12]	Prospective, randomized, double-blind clinical trial	80	134	107	41/93	56.2	12	ΙΙ
Franke et al. (2000) [13]	Prospective, randomized controlled trial	72	60	56	14/46	58.3 (28–75)	6	ΙΙ

Characteristics of Included Studies

Five out of eight studies (62.5%) were prospective randomized control trials with a level II LoE, two out of eight studies (25.0%) were retrospective observational studies of prospectively collected data with a level III LoE, while one out of eight studies (12.5%) was a prospective observational study with a level III LoE. The mean Modified Coleman Score was 70.25 (out of 100) with scores ranging from 80 to 60 (out of 100), indicating a good methodological quality. The mean follow-up was 7.9 ± 2.2 months. Risk of bias was assessed using the Cochrane RoB 2.0 tool across five randomized controlled trials and the ROBINS-I tool across the remaining three non-randomized studies. Two out of five randomized control trials were judged low risk, while the remaining three raised some concerns in two out of five domains. All three non-randomized studies were classified as having a moderate risk of bias. The results of the risk of bias assessment are presented in Table 2 and Table 3.

**Table 2:** Risk of Bias (RoB) assessment of the included studies. RoB was judged using the RoB 2 tool for randomized controlled trials. Judgments were classified as low, some concerns, or high across the following five domains: D1 = randomization process; D2 = deviations from the intended interventions (effect of assignment); D3 = missing outcome data; D4 = outcome measurement; D5 = selection of the reported result. Overall risk of bias was rated low only if all domains were low; high if any domain was high; otherwise, some concerns.

Study	Randomization (D1)	Deviations (D2)	Missing data (D3)	Outcome measurement (D4)	Reporting (D5)	Overall RoB
Donaubauer et al. (2024) [10]	Low	Low	Low	Low	Low	Low
Annegret et al. (2013) [11]	Low	Some concerns	Low	Low	Some concerns	Some concerns
Franke et al (2007) [12]	Low	Low	Some concerns	Low	Some concerns	Some concerns
Franke et al (2000) [13]	Low	Low	Low	Low	Low	Low
Gaisberger et al. (2021) [16]	Low	Some concerns	Low	Some concerns	Low	Some concerns

**Table 3:** Risk of bias assessed using the Risk of Bias in Non-randomized Studies of Interventions (ROBINS-I) tool.

Study	Bias due to confounding	Bias in the selection of participants	Bias in the classification of interventions	Bias due to deviations from intended interventions	Bias in the measurement of outcomes	Bias in the selection of the reported result	Overall risk of bias
Van der Zee-Neuen et al. (2023) [14]	Moderate	Moderate	High	Low	Moderate	Low	Moderate
Van der Zee-Neuen et al. (2022) [15]	Moderate	Moderate	High	Low	Moderate	Low	Moderate
Rühle et al. (2018) [17]	Moderate	Moderate	Low	Low	Moderate	Low	Moderate

A total of 2.152 patients were initially included in the studies, while at the long-term follow-up, 1,831 patients were available (85.1%). The mean age of the patients was 56.5 years (range = 19-79 years).

Pain Outcomes

**Figure 2:**
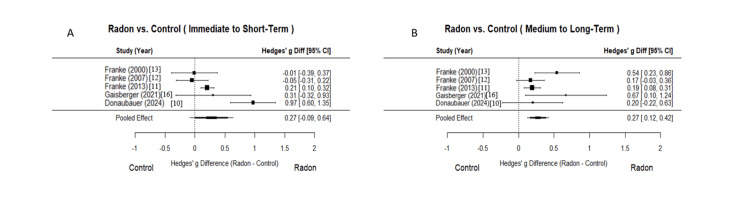
Forest plot of pain intensity following radon spa versus control sessions across time points. Panel A depicts the immediate to short-term results of radon spa treatment in pain intensity compared to control groups across five studies, with individual study effect sizes represented by Hedges’ g and 95% confidence intervals (CIs). The immediate to short-term comparison showed a non-significant pooled difference of 0.2748 (95% CI = -0.0888 to 0.6385, p = 0.1385), with high heterogeneity (I² = 86.90%, Q = 20.81, p = 0.0003). Panel B depicts the medium to long-term results of radon spa treatment in pain intensity compared to control groups across five studies, with individual study effect sizes represented by Hedges’ g and 95% CIs. For the radon spa treatment versus control comparison in the medium to long-term period, the pooled effect size difference was 0.2720 (95% CI = 0.1204 to 0.4237, p = 0.0004), indicating a statistically significant advantage of radon spa treatment over control, with low heterogeneity (I² = 43.28%, Q = 6.87, p = 0.1431).

The random-effects meta-analysis demonstrated a significant reduction in pain following radon spa treatment compared to control groups, with clinically meaningful improvements observed across two time points (Table 4, Figure 2). The meta-analysis of pain intensity revealed significant effects of radon spa treatment across two time periods. For the radon spa treatment versus control comparison in the medium to long-term period, the pooled effect size difference was 0.2720 (95% CI = 0.1204 to 0.4237, p = 0.0004), indicating a statistically significant advantage of radon spa treatment over control, with low heterogeneity (I² = 43.28%, Q = 6.87, p = 0.1431) across five studies. In contrast, the immediate to short-term comparison showed a non-significant pooled difference of 0.2748 (95% CI = -0.0888 to 0.6385, p = 0.1385), with high heterogeneity (I² = 86.90%, Q = 20.81, p = 0.0003).

**Table 4:** Meta-analysis results for radon versus control comparison and overall radon effect (pain intensity only).

Time point	Pooled g difference (radon vs. control), (95% CI)	I^2^	P-value	Overall radon effect (g) (95% CI)	Overall radon effect % change (95% CI)	I^2^	Sensitivity analysis	P-value
Immediate to short term	0.275 (-0.089, 0.638)	86.90%	0.139	0.966 (0.309, 1.624)	26.740 (14.391, 39.088)	98.89%	Consistent	0.004
Medium to long term	0.272 (0.120, 0.424)	43.28%	0.0004	0.994 (0.080, 1.907)	22.594 (8.124, 37.063)	99.55%	Consistent	0.033

**Figure 3:**
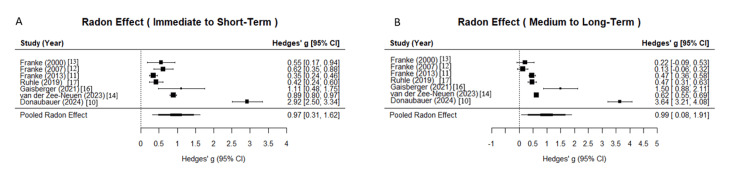
Forest plot of radon effect following radon spa sessions across time points. Panel A depicts the immediate to short-term overall effects of radon therapy compared to baseline across seven studies, with individual study effect sizes represented by Hedges’ g and 95% confidence intervals (CIs). The overall radon spa treatment effect compared to baseline demonstrated a pooled Hedges’ g of 0.9665 (95% CI = 0.3088 to 1.6243, p = 0.004), indicating a statistically significant therapeutic effect of radon spa treatment in patients with musculoskeletal disorders (MSDs). Panel B depicts the medium to long-term overall effects of radon therapy compared to baseline across seven studies, with individual study effect sizes represented by Hedges’ g and 95% CIs. The overall radon spa treatment effect compared to baseline demonstrated a pooled Hedges’ g of 0.9936 (95% CI = 0.0801 to 1.9070, p = 0.033), indicating a statistically significant therapeutic effect of radon spa treatment in patients with MSDs.

For the overall radon spa treatment effect compared to baseline, the immediate to short-term period demonstrated a pooled Hedges’ g of 0.9665 (95% CI = 0.3088 to 1.6243, p = 0.004), indicating a statistically significant therapeutic effect of radon spa treatment in patients with MSDs based on seven studies (Table 4, Figure 3). The medium to long-term period showed a pooled Hedges’ g of 0.9936 (95% CI = 0.0801 to 1.9070, p = 0.033), indicating a statistically significant therapeutic effect of radon spa treatment in patients with MSDs based on seven studies. Both periods exhibited very high heterogeneity (I² = 98.89% and 99.55%, respectively, p < 0.0001 for both), suggesting substantial variability across studies. Leave-one-out sensitivity analyses confirmed the robustness of these pooled estimates, with ranges of 0.6088 to 1.0751 for the immediate to short-term period and 0.5023 to 1.1403 for the medium to long-term period, indicating no single study disproportionately influenced the results.

Discussion

Our meta-analysis showed that patients receiving radon spa treatment had better outcomes in terms of pain reduction in the medium to long-term period compared to patients who received water-based spa treatment without radon. To our knowledge, this is the first meta-analysis of the contemporary literature comparing radon spa to water-based spa treatments on patients with MSDs.

Similarly, a systematic analysis examining radon treatments (not only radon spa, but any type of radon treatments, such as radon speleotherapy and baths) on rheumatic diseases which was published in 2005 and included five clinical trials with 338 patients showed that radon treatment had better results in terms of pain reduction compared to control interventions at three months and six months after treatment [26]. In addition, neither our study nor the 2005 study by Falkenbach et al. showed any statistically significant pain reduction immediately after treatment with radon spa.

Mechanisms

The exact biological mechanism of action of radon spa through which pain relief is achieved remains mostly elusive. The small doses of emitted alpha radiation from the radioactive noble gas radon, which range between 0.2 and 0.5 mSv, are believed to induce the analgesic effects [13]. In radon spa treatments, radon enters the body mainly via the skin epithelium, while inhalation only plays a minor role as the heads of the patients remain outside the tub. In open bath tubes and radon galleries, radon is also absorbed via the lungs [9].

Alpha radiation particles are believed to activate adaptive cellular responses, which reduce inflammation and enhance tissue repair. According to Shehata et al., a significant increase in circulating transforming growth factor-beta 1 (TGFβ-1) was measured in patients suffering from ankylosing spondylitis after treatment with combined spa exercise including radon [27]. TGFβ, through its anti-inflammatory function, plays a role in the reduction of tissue inflammation and is suggested to be one of the possible mechanisms through which pain relief is achieved after radon spa [28].

Immunological mechanisms have also been proposed. Studies have shown that radon leads to a significant rise in the count of regulatory T cells, which persists for months after the application of radon spa. A decrease in the expression of the majority of activation markers of the immune cell populations, such as CD69 on monocytes and on T-helper cells, has also been observed after radon spa treatment [10]. Moreover, Deloch et al. reported that radon treatment in an animal model of rheumatoid arthritis led to improved clinical response and was associated with an increase in peripheral blood B cells and Interleukin-5 concentration [29].

Yamaoka et al. reported significantly increased β-endorphin and adrenocorticotropic hormone levels in osteoarthritis patients after a combined hyperthermia and radon inhalation, suggesting that a mitigation of pain by radon treatment might be mediated by β-endorphin [30,31]. These osteo-immunological and neuroendocrine adaptations may underlie the sustained analgesic and functional gains reported in long-term observational cohorts [16].

Limitations

Limitations of this study include those inherent in the approach to conducting a systematic review and meta-analysis. Due to our strict inclusion and exclusion criteria, only eight studies were selected for evaluation. Not all studies reported the same data points, and there were studies missing one or more of the variables we included in our analysis. As a result, there was variability in the overall pool of patients and MSDs that were evaluated for each outcome. This variation of total patients evaluated for each outcome must be considered when interpreting these results. Moreover, as studies published on radon spa therapy are based mainly on self-reported outcomes from patients, studies on a large patient sample for every MSD and a long-term follow-up are extremely difficult to conduct. Another limitation of this study is that our methodology was limited by potential publication bias.

## Conclusions

There is good-quality evidence to show that radon spa therapy significantly reduces pain in the medium to long term in patients suffering from MSDs. Based on these results, it is suggested that radon spa therapy can be an effective alternative to other conservative means in the management of MSDs. Thus, more well-designed clinical trials are required to examine the clinical outcome of radon spa therapy on specific degenerative and autoimmune MSDs, while more preclinical studies are required to highlight the mechanism of radon spa therapeutic action on relieving musculoskeletal pain.

## References

[1] (2025). World Health Organization. Musculoskeletal conditions. https://www.who.int/news-room/fact-sheets/detail/musculoskeletal-conditions.

[2] Woolf AD, Pfleger B (2003). Burden of major musculoskeletal conditions. Bull World Health Organ.

[3] Gómez-Galán M, Pérez-Alonso J, Callejón-Ferre ÁJ, López-Martínez J (2017). Musculoskeletal disorders: OWAS review. Ind Health.

[4] Buchanan WW, Hogan MG, Kean CA, Kean WF, Rainsford KD (2024). Surgery of joints. Inflammopharmacology.

[5] Kean WF, Kean CA, Hogan MG (2021). Osteoarthritis. Therapeutic Choices.

[6] Babatunde OO, Jordan JL, Van der Windt DA, Hill JC, Foster NE, Protheroe J (2017). Effective treatment options for musculoskeletal pain in primary care: a systematic overview of current evidence. PLoS One.

[7] Buchanan WW, Rainsford KD, Kean CA, Kean WF (2024). Treatment of rheumatic musculoskeletal disorders. Inflammopharmacology.

[8] Sukenik S, Buskila D, Neumann L, Kleiner-Baumgarten A, Zimlichman S, Horowitz J (1990). Sulphur bath and mud pack treatment for rheumatoid arthritis at the Dead Sea area. Ann Rheum Dis.

[9] Maier A, Wiedemann J, Rapp F (2020). Radon exposure-therapeutic effect and cancer risk. Int J Mol Sci.

[10] Donaubauer AJ, Becker I, Klein G (2024). Effects of serial radon spa therapy on pain and peripheral immune status in patients suffering from musculoskeletal disorders- results from a prospective, randomized, placebo-controlled trial. Front Immunol.

[11] Annegret F, Thomas F (2013). Long-term benefits of radon spa therapy in rheumatic diseases: results of the randomised, multi-centre IMuRa trial. Rheumatol Int.

[12] Franke A, Reiner L, Resch KL (2007). Long-term benefit of radon spa therapy in the rehabilitation of rheumatoid arthritis: a randomised, double-blinded trial. Rheumatol Int.

[13] Franke A, Reiner L, Pratzel HG, Franke T, Resch KL (2000). Long-term efficacy of radon spa therapy in rheumatoid arthritis--a randomized, sham-controlled study and follow-up. Rheumatology (Oxford).

[14] van der Zee-Neuen A, Fuchs J, Wildburger S (2023). Improvement of pain symptoms in musculoskeletal diseases after multimodal spa therapy in the Austrian Gastein Valley-a study based on longitudinal registry data. Int J Public Health.

[15] van der Zee-Neuen A, Strobl V, Dobias H (2022). Sustained improvements in EQ-5D utility scores and self-rated health status in patients with ankylosing spondylitis after spa treatment including low-dose radon - an analysis of prospective radon indication registry data. BMC Musculoskelet Disord.

[16] Gaisberger M, Fuchs J, Riedl M (2021). Endogenous anandamide and self-reported pain are significantly reduced after a 2-week multimodal treatment with and without radon therapy in patients with knee osteoarthritis: a pilot study. Int J Biometeorol.

[17] Rühle PF, Klein G, Rung T (2019). Impact of radon and combinatory radon/carbon dioxide spa on pain and hypertension: results from the explorative RAD-ON01 study. Mod Rheumatol.

[18] Page MJ, McKenzie JE, Bossuyt PM (2021). The PRISMA 2020 statement: an updated guideline for reporting systematic reviews. BMJ.

[19] Coleman BD, Khan KM, Maffulli N, Cook JL, Wark JD (2000). Studies of surgical outcome after patellar tendinopathy: clinical significance of methodological deficiencies and guidelines for future studies. Victorian Institute of Sport Tendon Study Group. Scand J Med Sci Sports.

[20] Sterne JA, Savović J, Page MJ (2019). RoB 2: a revised tool for assessing risk of bias in randomised trials. BMJ.

[21] Sterne JA, Hernán MA, McAleenan A, Reeves BC, Higgins JP (2019). Assessing risk of bias in a non-randomized study. Cochrane Handbook for Systematic Reviews of Interventions Version 6.1.

[22] (2025). Oxford Centre for Evidence-Based Medicine. Levels of Evidence. https://www.cebm.ox.ac.uk/resources/levels-of-evidence/ocebm-levels-of-evidence.

[23] Keitel W, Hoffmann H, Weber G, Krieger U (1971). [Evaluation of the percentage of functional decrease of the joints using a motor function test in rheumatology]. Dtsch Gesundheitsw.

[24] (1990). EuroQol--a new facility for the measurement of health-related quality of life. Health Policy.

[25] Jaeckel WH, Cziske R, Schochat T, Jacobi E (1986). Assessing health status after inpatient rehabilitation in rheumatoid arthritis. Int Rehabil Med.

[26] Falkenbach A, Kovacs J, Franke A, Jörgens K, Ammer K (2005). Radon therapy for the treatment of rheumatic diseases--review and meta-analysis of controlled clinical trials. Rheumatol Int.

[27] Shehata M, Schwarzmeier JD, Hilgarth M (2006). Effect of combined spa-exercise therapy on circulating TGF-beta1 levels in patients with ankylosing spondylitis. Wien Klin Wochenschr.

[28] Kullmann M, Rühle PF, Harrer A (2019). Temporarily increased TGFβ following radon spa correlates with reduced pain while serum IL-18 is a general predictive marker for pain sensitivity. Radiat Environ Biophys.

[29] Deloch L, Hehlgans S, Rückert M (2022). Radon improves clinical response in an animal model of rheumatoid arthritis accompanied by increased numbers of peripheral blood B cells and interleukin-5 concentration. Cells.

[30] Yamaoka K, Mitsunobu F, Hanamoto K, Shibuya K, Mori S, Tanizaki Y, Sugita K (2004). Biochemical comparison between radon effects and thermal effects on humans in radon hot spring therapy. J Radiat Res.

[31] Yamaoka K, Mitsunobu F, Kojima S, Shibakura M, Kataoka T, Hanamoto K, Tanizaki Y (2005). The elevation of p53 protein level and SOD activity in the resident blood of the Misasa radon hot spring district. J Radiat Res.

